# Effectiveness of airport screening at detecting travellers infected with novel coronavirus (2019-nCoV)

**DOI:** 10.2807/1560-7917.ES.2020.25.5.2000080

**Published:** 2020-02-06

**Authors:** Billy J Quilty, Sam Clifford, CMMID nCoV working group, Stefan Flasche, Rosalind M Eggo

**Affiliations:** 1 Centre for the Mathematical Modelling of Infectious Diseases, Department of Infectious Disease Epidemiology, London School of Hygiene and Tropical Medicine, London, United Kingdom; 2 The members of the Centre for the Mathematical Modelling of Infectious Diseases (CMMID) nCoV working group are listed at the end of the article; 3 These authors contributed equally to this work

**Keywords:** 2019-nCoV, airport screening, effectiveness, thermal scanning, emerging infections, interventions, surveillance

## Abstract

We evaluated effectiveness of thermal passenger screening for 2019-nCoV infection at airport exit and entry to inform public health decision-making. In our baseline scenario, we estimated that 46% (95% confidence interval: 36 to 58) of infected travellers would not be detected, depending on incubation period, sensitivity of exit and entry screening, and proportion of asymptomatic cases. Airport screening is unlikely to detect a sufficient proportion of 2019-nCoV infected travellers to avoid entry of infected travellers.

As at 4 February 2020, 20,471 confirmed cases of novel coronavirus (2019-nCoV) have been reported from China with 425 deaths confirmed so far [1]. There were cases in at least 23 other countries, identified because of symptoms and recent travel history to Hubei province, China. This strongly suggests that the reported cases constitute only a small fraction of the actual number of infected individuals in China [2]. While the most affected region, Hubei province, has ceased air travel and closed major public transport routes [3] the number of exported cases are still expected to increase [4].

Despite limited evidence for its effectiveness, airport screening has been previously implemented during the 2003 SARS epidemic and 2009 influenza A(H1N1) pandemic to limit the probability of infected cases entering other countries or regions [5-7]. Here we use the available evidence on the incubation time, hospitalisation time and proportion of asymptomatic infections of 2019-nCoV to evaluate the effectiveness of exit and entry screening for detecting travellers entering Europe with 2019-nCoV infection. We also present an online tool so that results can be updated as new information becomes available.

## Simulation of travellers at each stage of infection with 2019-nCoV

We simulated 100 2019-nCoV infected travellers planning to board a flight who would pose a risk for seeding transmission in a new region. The duration of travel was considered as the flight time plus a small amount of additional travel time (ca 1 hour) for airport procedures. We assumed that infected individuals will develop symptoms, including fever, at the end of their incubation period (mean 5.2 days (Table)) [8] and progress to more severe symptoms after a few days, resulting in hospitalisation and isolation. We also took into account that individuals may have asymptomatic (subclinical) infection that would not be detected by thermal scanning or cause them to seek medical care, although these individuals may be infectious, and that infected travellers may exhibit severe symptoms during their travel and be hospitalised upon arrival without undergoing entry screening. We then estimated the proportion of infected travellers who would be detected by exit and entry screening, develop severe symptoms during travel, or go undetected, under varying assumptions of: (i) the duration of travel; (ii) the sensitivity of exit and entry screening; (iii) the proportion of asymptomatic infections; (iv) the incubation period and (v) the time from symptom onset to hospitalisation (Table).

**Table:** Parameter values and assumptions for the baseline scenario estimating effectiveness of exit and entry screening at airports for detecting passengers infected with novel coronavirus (2019-nCoV)

Parameter	Value (baseline scenario)	Source
Duration of travel	12 hours	Beijing – London [18]
Sensitivity of exit screening	86%	Sensitivity of infrared thermal image scanners [19]
Sensitivity of entry screening	86%	Sensitivity of infrared thermal image scanners [19]
Proportion of asymptomatic infections undetectable by typical screening procedures	17%	1 of 6 reported asymptomatic in a 2019-nCoV family cluster [11]
Incubation period	Mean 5.2 days, variance 4.1 days	Reported Gamma distributed mean, variance estimated from uncertainty interval of mean [8]
Time from symptom onset to hospitalisation	Mean 9.1 days, variance 14.7 days	Reported Gamma distributed mean, variance estimated from uncertainty interval of mean [8]

**Figure 1:**
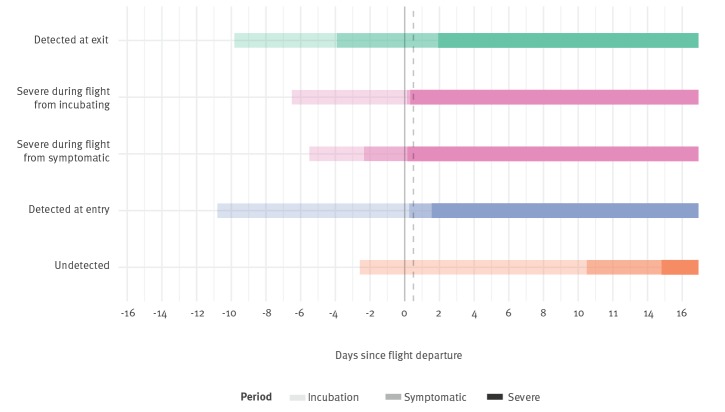
Simulated infection histories of travellers infected with novel coronavirus (2019-nCoV)

We assume that the time of starting travel is randomly and uniformly distributed between the time of infection and twice the expected time to severe disease, ensuring that simulated travellers are travelling during their incubation period. However, we only consider those travellers who depart before their symptoms progress to being so severe that they would require hospital care [8]. We simulate travellers with individual incubation period, time from onset to severe disease, flight start times and detection success at exit and entry screening according to the screening sensitivities (Figure 1). An individual will be detected at exit screening if their infection is symptomatic i.e. has detectable fever, their departure time exceeds their incubation period, and their stochastic exit screening success indicates detection. An individual will be detected at entry screening if their infection is symptomatic, their incubation period ends after their departure but before their arrival, they have not been detected at exit screening, and their entry screening result is positive despite imperfect sensitivity. Entry screening detections are further divided into detection due to severe symptoms and detection of mild symptoms via equipment such as thermal scanners. We used 10,000 bootstrap samples to calculate 95% confidence intervals (CI).

The incubation period begins on infection and travellers then progress to being symptomatic and having severe symptoms. Travellers may fly at any point within the incubation or symptomatic phases; any would-be travellers who show (severe) symptoms and are hospitalised before exit. Vertical lines represent the exit screening at start of travel (solid) and entry screening at end of travel (dashed) 12 hours later.

**Figure 2:**
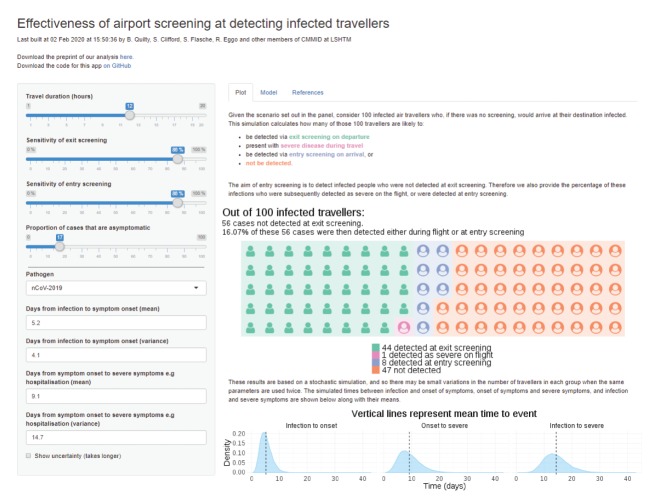
Screenshot of Shiny app^a^ displaying the number of travellers infected with novel coronavirus (2019-nCoV) detected at airport exit and entry screening with baseline assumptions^b^, 95% bootstrap confidence intervals, time distributions for incubation period and time to severe disease*

^a^ Source [9].

^b^ Baseline assumptions according to the Table.

The model code is available via GitHub [9] and the results can be further explored in a Shiny app [10] at https://cmmid-lshtm.shinyapps.io/traveller_screening/ (Figure 2).

Results are from stochastic simulation, and so there may be small variations in the number of travellers in each group when the same parameters are used twice. Sliders are provided to modify the duration of travel, the sensitivity of both exit and entry screening, the proportion symptomatic, and the natural history parameters for the infection.

## Effect of screening on detection

For the baseline scenario we estimated that 44 (95% CI: 33–56) of 100 infected travellers would be detected by exit screening, no case (95% CI: 0–3) would develop severe symptoms during travel, nine (95% CI: 2–16) additional cases would be detected by entry screening, and the remaining 46 (95% CI: 36–58) would not be detected.

**Figure 3:**
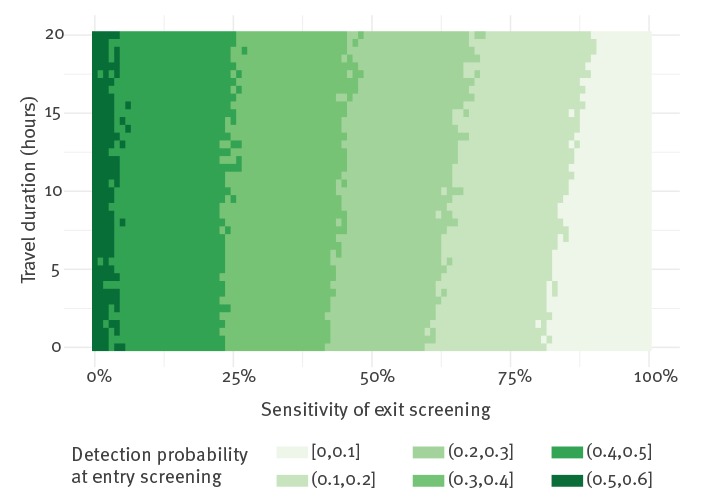
Probability of detecting travellers infected with novel coronavirus (2019-nCoV) at airport entry screening by travel duration and sensitivity of exit screening

The effectiveness of entry screening is largely dependent on the effectiveness of the exit screening in place. Under baseline assumptions, entry screening could detect 53 (95% CI: 35–72) instead of nine infected travellers if no exit screening was in place. However, the probability of developing symptoms during the flight increases with flight time and hence exit screening is more effective for longer flights (Figure 3).

Each cell is a mean of 10,000 model simulations. Other parameters (incubation period, symptom onset to hospitalisation period, and proportion of asymptomatic infections) were fixed at baseline assumptions (Table). Intervals are probabilities of detection, binned at increments of 10% (0–10%, 10–20%, etc.).

Syndromic screening designed to prevent infected and potentially infectious cases entering a country undetected is highly vulnerable to the proportion of asymptomatic infections and long incubation periods. If our baseline scenario is modified to have 0% asymptomatic 2019-nCoV infections and 100% sensitivity of entry screening, the incubation period will need to be around 10-fold shorter than the period from symptom onset to severe disease (e.g. hospitalisation) in order to detect more than 90% of infected travellers that would not otherwise report illness at either exit or entry screening.

## Discussion and conclusions

As a response to the ongoing outbreak of the 2019-nCoV originating in Wuhan, exit screening has been implemented for international flights leaving China’s major airports. Thermal scanning, which can identify passengers with fever (high external body temperature), allows for passengers exhibiting symptoms of 2019-nCoV infection to be tested before they board a plane. Similarly, entry screening for flights originating in the most affected regions may be under consideration at airports in regions in and outside China. We estimate that the key goal of syndromic screening at airports - to prevent infected travellers from entering countries or regions with little or no ongoing transmission - is only achievable if the rate of asymptomatic infections that are transmissible is negligible, screening sensitivity is almost perfect, and the incubation period is short. Based on early data from Li et al. [8], 2019-nCoV appears to have a shorter incubation period than severe acute respiratory syndrome (SARS), and a higher rate of asymptomatic infections [11]. Under generally conservative assumptions on sensitivity, we find that 46 of 100 infected travellers will enter undetected.

Entry screening is an intuitive barrier for the prevention of infected people entering a country or region. However, evidence on its effectiveness remains limited and given its lack of specificity, it generates a high overhead of screened travellers uninfected with the targeted pathogen [5]. For example, when entry screening was implemented in Australia in response to the 2003 SARS outbreak, 1.84 million people were screened, 794 were quarantined, and no cases were confirmed [12]. While some cases of 2019-nCoV infection have been identified through airport screening in the current outbreak, our estimates indicate that likely more infected travellers have not been detected by screening.

It is important to note that our estimates are based on a number of key assumptions that cannot yet be informed directly by evidence from the ongoing 2019-nCoV outbreak. The current outbreak has spread rapidly and early evidence suggests that the average disease severity is lower than that of SARS. This may also suggest a substantial proportion of asymptomatic cases. A recent analysis of a family transmission cluster is based on a small sample size but one in six infections was asymptomatic [11]; this is a major impediment for the effectiveness of syndromic screening. However, if asymptomatic cases were not infectious they would not pose a risk for seeding infection chains on arrival. To allow easy adaptation of our results as new insight becomes available in the coming weeks, we developed a free interactive online tool, available at https://cmmid-lshtm.shinyapps.io/traveller_screening/.

While the most up-to-date data on the incubation period or the time until recovery from 2019-nCoV infection have been used in this analysis, these figures are likely to change over time as more data become available. Unless the incubation period is only a small fraction of the duration of infection in relation to that of symptomatic disease, and fever in particular, syndromic screening is likely to detect an insufficient fraction of infected cases to prevent local infections. In addition, the sensitivity of airport screening for the detection of 2019-nCoV has not been evaluated. However, we chose conservative estimates and show that with reduced sensitivity, the effectiveness of syndromic screening would further decline.

In many international airports, information is provided to travellers from affected regions recommending action if they develop symptoms on or after arrival [13-16]. Some countries, for example Japan, also require incoming passengers to complete forms detailing their past and future travel in order to aid tracing [17]. Due to the duration of the incubation period of 2019-nCoV infection, we find that exit or entry screening at airports for initial symptoms, via thermal scanners or similar, is unlikely to prevent passage of infected travellers into new countries or regions where they may seed local transmission.

## *Erratum

Figure 2 was replaced on 7 February 2020.

## Members of the Centre for the Mathematical Modelling of Infectious Diseases (CMMID) nCoV working group

Yang Liu, Charlie Diamond, W John Edmunds, Sebastian Funk, Amy Gimma, James D Munday, Hamish Gibbs, Nikos I Bosse, Sam Abbott, Timothy W Russell, Petra Klepac, Mark Jit, Joel Hellewell.
