# Erratum for Euro Surveill. 2020;25(5)

**DOI:** 10.2807/1560-7917.ES.2020.25.6.2002132

**Published:** 2020-02-13

**Authors:** Eurosurveillance editorial team

**Affiliations:** 1 European Centre for Disease Prevention and Control (ECDC), Stockholm, Sweden

In the article ‘Effectiveness of airport screening at detecting travellers infected with novel coronavirus (2019-nCoV)’ by Quilty et al., published on 6 Feb 2020, Figure 2 was replaced on 7 February 2020.

